# The New Educational Minute

**Published:** 1862-01

**Authors:** 


### ART. VII.—THE NEW EDUCATIONAL MINUTE.

THE most cursory reader of newspapers must for many weeks past have seen plentiful evidence that some violent and unexpected tempest has disturbed the placidity of the educational world. National schoolmasters, a body of men whose presence and influence are scarcely at all felt by the community in ordinary seasons, have been holding lengthy and almost violent meetings in numerous places, and have been encouraged by the clergy, by school managers, and by other men usually cautious in speech and decorous in behaviour, in flinging such epithets as unjust, dishonest, and revolutionary, against an important department of the government. The public at large know so little about national education, and are accustomed to turn with such decided aversion from any reference to it, that they are particularly liable to be misled by an energetic clamour upon the subject. They can hardly fail to believe in the existence of a fire, when the smoke is so plentiful and so dense; or to be persuaded that some actual injustice is either done or threatened, when the cries are so reiterated, so piteous, and so loud. It is fitting, therefore, that we should place before our readers a brief summary of the matters in dispute; the more especially that we have formed opinions adverse to the complainants upon all, or nearly all, the *principles* at issue, and think the new educational code calculated to be of great advantage to the nation. It may possibly require some alterations of detail to relieve unexpected points of friction; it may, and probably will, produce individual cases of hardship both to schools and teachers; it may even pinch pretty generally during a brief transition period; but we feel convinced that it is a step in the right direction, and that the almost unanimous wail of schoolmasters and managers, of the persons who have for many years held an important trust that they have generally failed to discharge, should be regarded with the most grave suspicion and distrust. We are the more bound to defend the Revised Code against its numerous and powerful adversaries, inasmuch as its provisions are a practical embodiment of recommendations urged in former numbers of this journal, before the report of the Royal Commission was made public.* Before proceeding to consider the probable effect of these provisions, it is expedient to glance briefly at the system now in operation, and for this purpose we subjoin an extract from the *Saturday Review*, containing a concise but clear exposition of the rules that will be superseded, if Parliament should confirm, without very material change, the document known as the "Revised Code."

"At present, schools are supported from three different sources. These are the private subscriptions of benevolent persons; the weekly pence paid by the parents of the children attending the various schools; and, lastly, certain grants made out of the money voted annually by Parliament for Public Education, and distributed by that branch of the Privy Council called the Education Department. This department is guided by certain definite rules, which have been promulgated in the form of minutes; and the principles upon which these minutes have hitherto been framed may be thus described. In the first place, the general object of granting State aid at all has been to raise the standard of education throughout the country. Assistance, therefore, has been absolutely refused, except in cases where there was reasonable ground to believe that the education furnished would be of an efficient character. And for this purpose, certain conditions as to the school-buildings, and as to the teachers, have been considered indispensable. In the second place, the State assistance has been conveyed through some religious channel. Recognising the popular opinion that religion is an essential part of education, the Education Department has consulted the various religious bodies, and has refused to assist any school unconnected with some denomination, or in which some portion of the Holy Scriptures does not form part of the daily teaching. It appears that at least six or seven denominations have derived aid for the schools in connexion with them. These two principles always have been, and still continue to be, the cardinal principles of the State system of education in this country. Assuming them, therefore, the Education Department has, in the words of the Code, promoted the education of children belonging to the classes who support themselves by manual labour, by assisting voluntary efforts to build schools and to maintain them. Passing by the grants made for building, and numerous minor contributions for books and other apparatus, it may be said that the assistance has been furnished to existing schools in three different ways. In the first place, any teacher holding a certificate from the government is entitled to a certain sum by way of augmentation to his salary. In the second place, certain young persons apprenticed to certificated masters are entitled during their five years' apprenticeship to a certain stipend. In the third place, a capitation grant is made by the govern-

* See *Journal of Psychological Medicine* for 1859. Articles on "Artificial Production of Stupidity in Schools," and "Principles of Early Mental Education."

ment to schools, in proportion to the number of children attending school during 176 days in the year. But beside these several modes of aiding schools, the government contributes very largely to support normal schools for training future masters and mistresses. The number of these institutions is now 33; and, in some cases, as in that of Cheltenham, the government contributes 94 or 95 per cent. of the whole annual expenditure. Sometimes in the form of Queen's Scholarships, which are held by meritorious students, sometimes in the form of salaries to lecturers. As an instance of the way in which the Parliamentary Grant is spent, take the year 1859. Out of a total sum of 723,115*l.* the sum of 86,328*l.* was spent in augmenting salaries, the sum of 252,550*l.* in the stipends of pupil teachers, and in gratuities to certificated teachers for instructing them; the sum of 89,587*l.* upon training colleges; whilst the sum of 61,183*l.* was expended in capitation grants. It is obvious, therefore, that the expenditure upon teachers constitutes the largest item in the account."

Now it is urged, by the advocates of the above described system, that it has already produced very beneficial results; and they support their view by statistics showing that the numerical proportion of scholars to the population in England and Wales, is larger than either in France or Holland; although, a few years ago, it was very much smaller. Furthermore, the system has produced a vast number of educated teachers; and, lastly, it has enlisted all the great religious bodies in the work of education, by permitting them to teach their respective tenets in their own schools, without let or hindrance. On the other side of the question it has been said that the mere attendance of children at schools does not necessarily involve education; that the production and payment of teachers would be better left to the operation of the ordinary rules of supply and demand; and that the activity of persons of extreme religious views often produces some kind of distrust, or at least want of cordiality, on the part of those of more moderate opinions; who, after all, make up the great bulk of the tax-paying community. The New Code may be held, we think, to be a practical expression of the latter notions; and, the children having been already brought to the water, it is, in fact, an attempt to make them drink.

A young lady reared in the midland coal district, being questioned concerning her educational history, replied that she had "bin t'neet school onst, but there wor noa candles, and t'missis didn't come." Now it is perhaps not fair to say that this "attendance" would have counted towards a claim for a capitation grant; but it is certain that attendances equally perfunctory are so counted daily, and that the results of elementary school teaching have been shamefully and notoriously inadequate to the labour and money wasted in procuring them. The methods of teaching are often as bad as bad can be, the subjects taught are far too numerous and too abstruse for the time and mental capacity of the scholars, and the general tendency of the system has been to make the master neglect the foundation of all learning, the right instruction of the junior classes, in order to cram the seniors with lessons not only undigested, but usually indigestible. The report of the Education Commission was not needed in order to enable us to condemn the quality of the teaching—for Her Majesty's Inspectors had already borne testimony to it. The terrible piece of writing placed on record by Mr. Brookfield,* and the opinions of Mr. Watkins, Mr. Kennedy, and Dr. Woodford, told, in 1856, the same tale that has since been so loudly repeated. The educational grant for last year amounted to £800,000, and was distributed among 8000 schools. There are 15,000 other schools, now in existence, of a kind that will be certain or likely to claim assistance, and to fulfil the conditions for demanding it. The increase in the grant, regularly progressive hitherto, would soon require a sum that would be counted in millions; and it has become impossible to expend more money upon a system of teaching so miserably barren of results, and the barrenness of which depends, moreover, upon demonstrable errors of principle in the methods pursued. Space does not allow us to dwell upon this aspect of the subject on the present occasion; but in the two essays already referred to, we endeavoured to point out both the violation of natural laws, and the inevitable practical results of such violation, which form parts of our present method of national education.

For the future, under the New Code, instead of three methods of aiding schools, there will be only one. The managers will receive a certain fee for every child who has attended school for a specified number of days, and who can pass an examination, satisfactory to the Inspector, in reading, writing, and arithmetic. Failure in any one of these will forfeit one-third; in any two two-thirds; and in any three, the whole of the fee in question. For the purpose of examination, the children are to be classified

---

* See *Journal of Psychological Medicine*, April, 1859. "Mr. Brookfield called upon two children aged about eleven years, 'who did their arithmetic and reading tolerably well, who wrote something pretty legible, intelligible, and sensible about an omnibus and about a steam-boat,' to write down the answers of the Church catechism to two questions. It must be observed that they had been accustomed to *repeat* the Catechism during half an hour of each day, in day-school and Sunday-school, for four or five years, and the following is an example of what they wrote:—

"'My duty toads God is to bleed in him to fering and to loaf withold your arts withold my mine withold my sold and with my sernth to whirchp and to give thinks to put my old trast in him to call upon him to onner his old name and his world and to save him truly all the days of my lifes end.'"

For further illustrations see the Journal referred to, pp. 198, 199.

according to ages; and from each class a different standard will be required.

The principle of this regulation is, we think, evidently good; and the selection of the three subjects named as the only test-subjects, most judicious.

To have these essentials well taught, to have no temptation to slur them over in order to trace out the course of the Euphrates, or to commit to memory the genealogy of the Pharaohs, will very greatly stimulate the intellectual activity of the children of the poor. Their more advanced reading lessons may then be made the vehicles of real knowledge; or, in the words of the *Daily Telegraph*, "Reading, taught out of the right books, will teach geography and history; writing is the alphabet of form; and arithmetic may be pursued to the very borders of mathematics."

In order to obtain these benefits, and in order to carry out the examination test with fairness and efficiency, it will be necessary that School Inspectors should be selected from men not only diligent, careful, and conscientious in the discharge of their duties, but conversant with the mental operations of children, and able to gauge the degree in which the methods of teaching used have succeeded in reaching and arousing the intelligence of the pupils. It will be found necessary, also,—and we are glad to learn from private sources that it is intended,—to modify that part of the Code which provides for the examination of very young children. We cannot approve of teaching the three test-subjects sooner than at seven or eight years of age; and think that the aim of infant schools for the poor should chiefly be to furnish their children with stimuli to mental activity, analogous to those furnished unconsciously, higher in the social scale, by the pursuits and conversation of friends and parents. Infant schools might, in this way, be of great utility; and their utility might easily be tested,—but not, we apprehend, by an examination of babies in reading, writing, and arithmetic.

The withdrawal of government aid from masters, mistresses, and pupil teachers, is one of the points at which there is reason to anticipate hardship in individual cases. It will be very generally conceded that the Normal School system has produced a very bad article—a teacher crammed to repletion with lip knowledge of a kind useless in his vocation, and burning with a fierce desire to impart it, or the phantasm or outward emblem of it, to all under his charge. A master fresh from a training college, in a country parish, is a phenomenon to be regarded with profound and respectful admiration. If he did but know how to teach children to read, he would, like the venerable ex-Chancellor, know a little of everything. If he be naturally foolish, he becomes a standing example, to the villagers, of the inutility of what appears to them to be learning. If he be intelligent, he is very likely to pine for some more conspicuous field of action, to become discontented with his humble lot, and careless in the performance of his humble duties.

The part of the New Code which makes the higher classes of certificates the reward, not of examinations in useless knowledge, but of specified periods of successful teaching, is likely to do much to keep the heart of the master in his work. To teach children well, will hereafter be the road to a higher position and larger stipend. We say larger stipend, because, although the certificates will no longer carry a government annuity in aid of salary, they will certainly carry a definite value in money, and will be sought for by the managers of the richer schools. The so-called salaries are now absurdly low, because they are practically increased by the national grant. The grant being withdrawn, the teacher, then as now, will require the market price of his labour, and his salary will be raised. This is, we think, self-evident, although the new arrangement will require time for its completion, and may possibly involve hardship while it is incomplete.

The working of the New Code, in financial matters, is a subject on which much has been written, probably to very little purpose. We have seen many estimates, official and private; but we have seen scarcely two alike; and it is probable that none of them are entirely trustworthy. In the case of a girls'-school, extremely well conducted, an examination was made by the ladies of the school committee, without any previous notice either to teachers or scholars, expressly as a means of estimating the future prospects of the school as far as pecuniary aid from government was concerned. The results, which were verified by a second examination conducted by different persons, may be stated in the following manner:—

| | |
|---|---|
| Number of scholars on the books . . . . . . . | 193 |
| Average daily attendance for the last school year . | 169 |
| Number of scholars who fulfilled the conditions of the new minute, with regard to attendance, during the year preceding the last visit of Her Majesty's Inspector . . . . . . . . . . . . . . . . | 163 |
| Aggregate number of attendances, above 100, of these scholars—being 169 days, or 338 times, for each scholar . . . . . . . . . . . . . . . . | 38,894 |

Scholars present in the school on the 9th of October, 1861, (the day of the unexpected examination), classified into groups by age, as required in the new minute:—

| | | |
|---|---|---|
| Group | 1 | 47 |
| " | 2 | 38 |
| " | 3 | 61 |
| " | 4 | 32 |
| | Total | 178 |

Failures in each group, when examined in accordance with the new minute :—

| | | Reading. | Writing. | Arithmetic. |
|---|---|---|---|---|
| Group | 1 | 5 | 6 | 15 |
| " | 2 | 2 | 26 | 10 |
| " | 3 | 2 | 31 | 54 |
| " | 4 | 2 | 12 | 19 |
| | | 11 | 75 | 98 |

In the arithmetical exercises, each exercise containing *one* error was considered as a failure. In writing from dictation, *one* error, and, in a few cases, *two*, in the spelling were passed as correct. The amount received from Government last year, in all ways was 114*l.* 10s. Under the new minute, according to the foregoing examination, the amount would be from 110*l.* to 120*l.*

We strongly suspect, therefore, that in all those cases where masters and managers expect to lose half their present aid, the expectation shows that they have not done half their duty. If, however, the New Code should be found seriously to diminish the present income of schools, there will be no lack of compensating influences. It cannot be doubted that the examinations of the first year will be indulgent; the Inspectors showing what they will in future require, rather than being extreme to mark deficiencies. The better teaching reasonably to be expected will probably increase the subscriptions of the benevolent, and, furthermore, it would be plainly necessary for the Privy Council to increase the amount of the capitation fee, if it were found by experience to be insufficient. This is the very point on which the value of conjecture can only be shown by trial ; and we trust that Parliament will be found ready to accept the Code, subject to such alterations as its framers may themselves suggest, after they have weighed the various objections hitherto advanced against it. We confidently believe that the principles on which the Code is based, will effect a radical and much-needed reform in the whole system of National Education ; and that this reform will rapidly spread into independent or adventure schools, and will leaven classes of society placed much above that in which it will originate.

